# Maternity leave and breastfeeding during COVID: a cross-sectional study

**DOI:** 10.1186/s13006-025-00779-5

**Published:** 2025-11-19

**Authors:** Julianne Miller, Patrick T. Bradshaw, Lia C. H. Fernald

**Affiliations:** 1 Division of Community Health Sciences, School of Public Health, University of California, 2121 Berkeley Way West, Berkeley, CA 94704 USA; 2 Division of Epidemiology, School of Public Health, University of California, Berkeley, USA

**Keywords:** COVID-19, Breastfeeding, Maternity leave, Paid leave

## Abstract

**Background:**

Maternity leave is an important driver of a woman’s ability to breastfeed, but this association may have changed during the COVID pandemic, which introduced new employment dynamics and breastfeeding challenges for mothers in the workforce. Our objectives were to examine the associations between maternity leave length and type of maternity leave (paid vs. unpaid) with breastfeeding initiation and breastfeeding duration during the pandemic, and to compare our findings with pre-pandemic data.

**Methods:**

Our sample was 3,683 recently postpartum women, currently in the workforce, who had given birth between March-December 2020 in the US. Data were obtained from the Pregnancy Risk Assessment and Monitoring System (PRAMS) survey, a population-based surveillance system developed by the Centers for Disease Control (CDC) in the US. We examined associations between taking longer maternity leave compared with shorter (*≥* 3 vs. < 3 months), and breastfeeding initiation or continuation (at 1, 2, and 3 months after delivery). We also examined heterogeneity in the associations between maternity leave and breastfeeding by a range of characteristics.

**Results:**

During COVID, having a longer maternity leave was not associated with breastfeeding initiation or continuation. These results contrast with pre-pandemic findings from the same cohort, in which a longer maternity leave was associated with more breastfeeding. In tests of heterogeneity in our current analysis, women who were younger, not married, less educated, or who did not receive any pay during their leave were less likely to continue breastfeeding if they took a shorter maternity leave than if they took a longer maternity leave.

**Conclusions:**

The COVID pandemic created a natural experiment for exploring associations between employment and breastfeeding among women in the workforce. Our results suggest that the conditions of the pandemic may have minimized average differences in breastfeeding outcomes by maternity leave length because more women were at home. However, our study highlighted that benefits were concentrated among women who were older, more educated, married, and who received paid leave, reinforcing the urgent need to support women who face greater economic and social disadvantages within the US.

**Supplementary Information:**

The online version contains supplementary material available at 10.1186/s13006-025-00779-5.

## Background

Breastfeeding is internationally recognized as a significant public health intervention given its health benefits for both mothers and infants. Exclusive breastfeeding for the first 6 months of life and continued breastfeeding for the first year of life and beyond are standard recommendations [1–3], and yet the United States falls well short of the breastfeeding goals recommended in Healthy People 2030 [4]. There are many barriers to breastfeeding including lack of adequate maternity leave for women in the workforce, which makes it difficult to breastfeed [5–13]. Returning to work within 6 weeks of birth – standard in the United States – is strongly associated with breastfeeding cessation, as is returning to work 6–12 weeks after birth [11]. Key factors associated with longer breastfeeding include having an occupation with flexible work schedules and minimizing mother/baby separation [10].

Paid maternity leave can be an important driver of a woman’s ability to breastfeed [14], and women who receive 12 or more weeks of paid maternity leave compared with women without paid maternity leave have shown greater initiation and continuation of breastfeeding [15]. Taking a shorter maternity leave (< 3 months) has been associated with a lower prevalence of ever breastfeeding and any breastfeeding at 1,2, or 3 months compared to a taking a longer maternity leave (*≥* 3 months) [16]. Physically returning to work in-person after childbirth, especially during the first three months, negatively impacts breastfeeding outcomes among employed women [9–12, 17–22]. Effects of shorter maternity leave length on breastfeeding cessation has been shown to be strongest among non-managers and in occupations with inflexible schedules [11]. Similarly, flexible work schedules and part-time work are factors that have been identified as facilitators of breastfeeding among employed women [13, 20, 23].

Associations of employment and breastfeeding duration are not equal across all occupations, and women who hold administrative or manual labor jobs with rigid schedules and little flexibility are more likely to stop breastfeeding upon returning to work when compared with stay-at-home mothers [13]. There are no differences in breastfeeding duration between women with professional level jobs and stay-at-home mothers, which may be explained by the high level of flexibility and control over time and schedules shared by women in professional level jobs and stay-at-home mothers [13]. Thus, having a flexible work schedule, along with having proximity to the baby with a private place to feed or access to pump, may be the most important factors for supporting sustained breastfeeding among employed women [13].

Previous analyses using data from the Pregnancy Risk Assessment and Monitoring System (PRAMS) survey [24], a population-based surveillance system developed by the Centers for Disease Control and Prevention (CDC), have shown that women who took shorter maternity leaves (< 3 months) were less likely to initiate and continue breastfeeding than women who took longer maternity leaves (*≥* 3 months) [16]. Beginning in 2020, the COVID-19 pandemic introduced new employment dynamics and breastfeeding challenges for mothers in the workforce, however, which may have changed these associations [25]. To explore whether the associations between maternity leave and likelihood of breastfeeding remained consistent during COVID, we examined data from March 2020 to December 2020. Our specific objectives were to examine the associations between maternity leave length and type of maternity leave (paid vs. unpaid) with breastfeeding initiation and breastfeeding duration at 1, 2, and 3 months after delivery during the COVID-19 pandemic; and to describe how the findings compare to data collected prior to the pandemic.

## Methods

The data for our analyses were drawn from PRAMS [24], which collects data about pregnancy, childbirth, and the postpartum period from a diverse sample of women across the United States. We analyzed standardized PRAMS data from sites that used both core and optional standard questions, which allowed for us to answer our research questions; we only included sites with a weighted response rate of at least 55%. We used data from the eight PRAMS sites (Massachusetts, Maryland, Missouri, New Hampshire, Oregon, Vermont, Wisconsin, and New York City) that included employment questions on their questionnaire and met our selection criteria, including women who provided information on maternity leave, prenatal employment, length of their maternity leave, and whether the leave was paid or unpaid. All participants were asked whether they would be willing to provide their consent to participate in the study, in accordance with the Declaration of Helsinki. An informed consent document was provided to participants in the survey packet or during phone interviews, and consent was implied by completing the survey.

Our analytic sample included women who gave birth to a live infant between March and December 2020; were employed during pregnancy, returned or planned to return after delivery to the same job that they held during pregnancy, and were 18 or older.

Maternity leave was coded as paid or unpaid, and length was originally categorized as 0 to 5 weeks, 6 to 12 weeks, and 13 or more weeks in accordance with previous studies [16, 26], and later condensed to a binary variable. Any breastfeeding, which was defined as from the breast or feeding pumped breastmilk, was coded at 1, 2 or 3 months postpartum. Breastfeeding initiation was defined as ever directly breastfeeding or pumping breastmilk for feeding. Covariates were maternal factors, which included self-reported race and Hispanic origin, age, education, smoking, parity, marital status (defined by PRAMS as legally married), household income by federal poverty level, and the infant’s gestational age at birth; fixed effects were included for PRAMS site.

With logistic regression models using survey weights, we examined the associations between maternity leave length taken during the pandemic (< 3 months vs. *≥* 3 months) and any breastfeeding: initiation, at 1 month, at 2 months, and at 3 months. We estimated unadjusted prevalence ratios (PRs) and adjusted prevalence ratios (APRs) including all covariates listed above, and then ran separate models for women by paid leave status (any leave/no paid leave).

Using standard techniques to examine heterogeneity, we calculated unadjusted and adjusted interactions between maternity leave length and six selected characteristics (paid maternity leave, maternal education level, maternal race/Hispanic origin, poverty-income ratio, maternal age, and marital status). We also conducted a sub-group analysis (*n* = 1,530) to investigate if there was an interaction between maternity leave length and whether a woman reported being able to work from home during the pandemic [13, 19]. For all analyses, we used specific Stata protocols in our analyses provided by CDC PRAMS that include weighting, sampling, and stratification (Additional File 4).

## Results

**Fig. 1:**
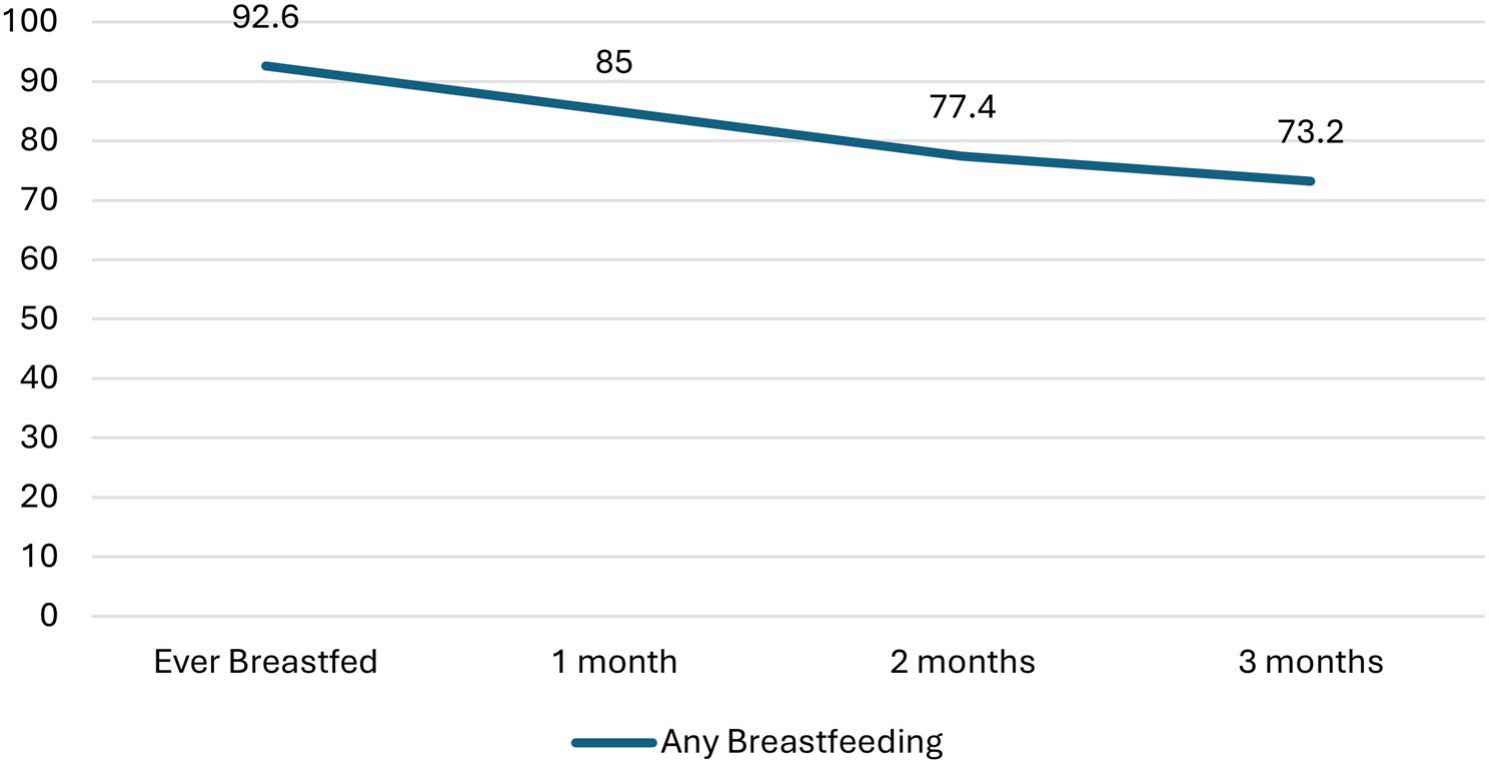
Percentage of babies receiving any breastmilk during the first 3 months, among children born in 2020

In our study of 3,683 recently postpartum women in the workforce who had given birth between March-December 2020, the percentage of women who reported taking any maternity leave was 94.4%. Likelihood of taking greater than or equal to a three-month maternity leave was associated with women having any paid leave, being ages 35 and older, primiparous, married, having a bachelor’s degree or higher, a household income level higher than 200% of the poverty line, or an infant born preterm (Table 1). About 40–50% of women took maternity leave of more than three months, although only about a third of Non-Hispanic White women took three or more months leave. Most women in our study reported ever breastfeeding their infants right after birth (92.6%), although breastfeeding rates decreased immediately afterwards. At one month, 85.0% of the women were breastfeeding, at two months, 77.4% reported any breastfeeding, and at three months, 73.2% reported any breastfeeding (Fig. 1).

**Table 1:** Descriptive statistics: Prevalence of maternity leave length after delivery among women in the United States during the COVID-19 pandemic by selected characteristics: Pregnancy Risk Assessment and Monitoring Systems (PRAMS), 8 US Sites, March-December, 2020

			Maternity Leave Length	
Characteristic	Level	Total^a^	< 3 months (%)	*≥* 3 months (%)
n(%)		3,683	2,202 (59.8)	1,481 (40.2)
Type of leave				
	No paid leave^b^	1,521	1,060 (69.7)	461 (30.3)
	Any paid leave	2,162	1,144 (52.9)	1,018 (47.1)
Maternal Race and Hispanic Origin				
	Hispanic	903	452 (50.1)	451 (49.9)
	Non-Hispanic White	1,480	943 (63.7)	537 (36.3)
	Non-Hispanic Black	470	241 (51.2)	229 (48.8)
	Non-Hispanic other	830	466 (56.2)	364 (43.8)
Maternal age, years	18–24	338	233 (68.8)	105 (31.2)
	25–29	850	541 (63.6)	309 (36.4)
	30–34	1,472	858 (58.3)	614 (41.7)
	35+	1,023	520 (50.8)	503 (49.2)
Maternal education	High School or less	651	435 (66.8)	216 (33.2)
	Some College or AS	879	544 (61.9)	335 (38.1)
	Bachelor’s degree or higher	2,153	1,231 (57.2)	922 (42.8)
Marital Status	Married	2,631	1,552 (59.0)	1,079 (41.0)
	Not married	1,052	654 (62.2)	398 (37.8)
Household income by federal poverty level, %	*≤* 100%	373	280 (75.2)	93 (24.8)
	100–200%	539	328 (60.9)	211 (39.1)
	> 200%	2,771	1,602 (57.8)	1,169 (42.2)
Parity	Primiparous	1,760	989 (56.2)	771 (43.8)
	Multiparous	1,923	1,215 (62.9)	708 (37.1)
Gestational age at birth	< 37 weeks (preterm)	533	302 (56.6)	231 (43.4)
	*≥* 37 weeks (term)	3,150	1,893 (60.1)	1,257 (39.9)
Site				
	Massachusetts	558	247 (44.3)	311 (55.7)
	Maryland	411	248 (60.3)	163 (39.7)
	Missouri	306	247 (80.8)	59 (19.2)
	New Hampshire	315	214 (67.8)	101 (32.2)
	Oregon	652	327 (50.2)	325 (49.8)
	Vermont	384	238 (62.1)	146 (37.9)
	Wisconsin	697	546 (78.3)	151 (21.7)
	New York City	361	150 (41.6)	211 (58.4)

^a^ Unweighted sample size

^b^ Includes women who took unpaid maternity leave or did not take any maternity leave

There were no differences in breastfeeding initiation rates based on having less than three months versus more than three or more months of maternity leave (91.9% vs. 93.7%; APR = 0.98; 95% CI 0.96, 1.01) (Table 2), and these results were similar at one, two and three months postpartum. Furthermore, there were no differences among women in their breastfeeding rates in terms of type of leave (paid v. unpaid) (Table 3). In tests of heterogeneity, women who were not married, younger, less educated, or who did not receive any paid maternity leave were less likely to continue breastfeeding if they took a shorter maternity leave (< 3 months) than if they took a longer maternity leave (*≥* 3 months) (Additional Files 1–3). No significant interactions were identified between maternity leave length and whether a woman reported being able to work from home even when accounting for whether the maternity leave was paid.

**Table 2:** Prevalence and prevalence ratio of breastfeeding initiation and breastfeeding at 1, 2, and 3 months by leave length among women with a recent live birth who were employed during and after pregnancy, Pregnancy Risk Assessment and Monitoring Systems (PRAMS), 8 U.S. Sites, March-December, 2020

	Total^a^	Unadjusted % (95% CI)^b^	Prevalence Ratio (95% CI)^d^	Adjusted % (95% CI)^b, c^	Adjusted Prevalence Ratio (APR) (95% CI)^c, d^
*Initiated breastfeeding*, *n = 3*,*683*					
*Leave length*					
< 3 months	2,119	91.1 (89.4, 92.9)	0.96 (0.94, 0.99)	91.9 (90.4, 93.5)	0.98 (0.96,1.01)
*≥* 3 months	1,564	94.7 (93.0, 96.3)	1 (Ref)	93.7 (91.8, 95.5)	1 (Ref)
*Breastfeeding at 1 month*,* n=*,* 3*,*683*					
*Leave length*					
< 3 months	2,119	83.1 (80.9, 85.4)	0.95 (0.91, 0.98)	84.0 (82.1, 86.0)	0.97 (0.93, 1.01)
*≥* 3 months	1,564	87.9 (85.5, 90.3)	1 (Ref)	86.7 (84.1, 89.3)	1 (Ref)
*Breastfeeding at 2 months*, *n = 3*,*683*					
*Leave length*					
< 3 months	2,119	75.2 (72.7, 77.8)	0.93 (0.89, 0.98)	76.3 (74.0, 78.5)	0.96 (0.92, 1.01)
*≥* 3 months	1,564	80.7 (77.8, 83.5)	1 (Ref)	79.3 (76.3, 82.2)	1 (Ref)
*Breastfeeding at 3 months*, *n = 3*,*026*^*e*^					
*Leave length*					
< 3 months	1,704	71.5 (68.6,74.5)	0.96 (0.90, 1.02)	73.0 (70.4, 75.7)	1.00 (0.94, 1.05)
*≥* 3 months	1,322	74.5 (71.1, 77.9)	1 (Ref)	73.3 (70.0, 76.7)	1 (Ref)

^a^Unweighted sample size

^b^Weighted % (95% CI)

^c^Adjusted for type of leave, maternal race, maternal age, maternal education, marital status, household income by federal poverty level, parity, gestational age at birth, timing of survey completion, and PRAMS site

^d^Marginal (averaged) prevalence ratios based on a logistic regression model

^e^Sample was restricted to those who had completed their PRAMS survey at or after 3 months after giving birth

**Table 3:** Prevalence and prevalence ratio of breastfeeding initiation and breastfeeding at 1, 2, and 3 months stratified by type of leave among women with a recent live birth who were employed during and after pregnancy, pregnancy high risk assessment monitoring system, 8 U.S. Sites, 2020–2021

Total^a^		No Paid Leave		Total^a^	Any Paid Leave		*p* ^g^
		Adjusted % (95% CI)^b, c^	APR (95% CI)^c, d,e^		Adjusted % (95% CI)^b, c^	APR (95% CI)^c, d,e^	
*Leave length*							0.225
< 3 months	983	91.0 (88.9, 93.2)	0.97 (0.93, 1.00)	1,136	93.1 (90.8, 95.4)	1.00 (0.96, 1.03)	
*≥* 3 months	538	94.2 (91.6, 96.8)	1 (Ref)	1,026	93.4 (90.8, 95.9)	1 (Ref)	
*Initiated breastfeeding*, *n = 1*,*521 Initiated breastfeeding*, *n = 2*,*162*							
*Leave length*							0.226
< 3 months	983	83.2 (80.4, 86.0)	0.95 (0.89, 1.00)	1,136	85.0 (82.1, 88.0)	0.99 (0.94, 1.04)	
*≥* 3 months	538	87.9 (84.1, 91.8)	1 (Ref)	1,026	85.9 (82.5, 89.3)	1 (Ref)	
*Breastfeeding at 1 month*, *n = 1*,*521 Breastfeeding at 1 month*, *n = 2*,*162*							
*Leave length*							**0.037**
< 3 months	983	77.4 (73.7, 81.1)	**0.91 (0.85**,** 0.97)**	1,136	81.0 (77.7, 84.3)	1.00 (0.94, 1.07)	
*≥* 3 months	538	85.0 (80.3, 89.6)	1 (Ref)	1,026	80.5 (76.7, 84.5)	1 (Ref)	
*Breastfeeding at 2 months*, *n = 1*,*521 Breastfeeding at 2 months*, *n = 2*,*162*							
*Leave length*							**0.005**
< 3 months	801	71.0 (67.0, 74.9)	**0.91 (0.83**,** 0.98)**	903	75.3 (71.6, 79.1)	1.07 (0.98, 1.15)	
*≥* 3 months	462	78.2 (73.1, 83.3)	1 (Ref)	860	70.7 (66.4, 74.9)	1 (Ref)	
*Breastfeeding at 3 months*, *n = 1*,*263*^*f*^ *Breastfeeding at 3 months*, *n = 1*,*763*^*f*^							

^a^ Unweighted sample size

^b^ Weighted % (95% CI)

^c^ Adjusted for type of leave, length of leave, maternal race, maternal age, maternal education, marital status, household income by federal poverty level, parity, gestational age at birth, timing of survey completion, and PRAMS site

^d^ We created separate survey-weighted multivariable logistic regression models to examine the association between type and length maternity leave and breastfeeding outcomes between those women who reported less than 3 months of maternity leave and those who reported 3 or more months of maternity leave. The reference group is women who took 3 months or more of maternity leave

^e^ Marginal (averaged) prevalence ratios based on a logistic regression model

^f^ Sample was restricted to those women who had completed their PRAMS survey at or after 3 months after giving birth

^*g*^ *P* value based on the Wald test of nonlinear combinations for an interaction between leave length and type of leave

## Discussion

We found no differences in breastfeeding initiation or duration by length of maternity leave during COVID, in contrast to previously published work on this same topic using the same dataset from the time period immediately pre-COVID with data from 2018 [16]. Comparable analyses using pre-pandemic data from the PRAMS cohort have shown associations between shorter maternity leave length and a lower prevalence of breastfeeding initiation and duration [10, 11, 16, 20, 27], but these findings were not maintained during the COVID-19 pandemic. When compared with a similar analysis using data from 2016 to 2018, a higher percentage of women in our study took a longer (*≥* 3 months) maternity leave (40.2% vs. 33.8%) and received paid leave (47.1% vs. 37.5%) [16]. A comparison of descriptive statistics between our study and the analysis of the earlier cohort suggests that although a higher percentage of women took longer maternity leaves and received paid maternity leave in 2020–2021 than in 2016–2018, the racial/ethnic, socioeconomic, and personal/social characteristics associated with paid leave remained consistent. Breastfeeding initiation rates were high (>90%) in both our study and in the equivalent pre-COVID analysis, and the prevalence of breastfeeding decreased over time in both studies. Breastfeeding appeared to have been slightly higher at each month in our study than in in the previous study (85.0% vs. 81.2% at 1 month, 77.4% vs. 72.1% at 2 months and 73.2% vs. 65.3% at 3 months), but we were not able to test these differences for significance.

A potential mechanism explaining the findings in our study could be that the COVID-19 pandemic introduced flexibility with work schedules and delayed the return to in-person work for many employed mothers. These factors have been shown to improve breastfeeding rates, and therefore may have balanced out the negative effects of a shorter maternity leave on breastfeeding outcomes. The mandated stay-at-home orders in the United States created a massive shift to work-from-home for an estimated 50% of paid workers in the United States between April and December 2020, compared with only 5% who were working from home prior to the pandemic [28]. The divergence to working from home is likely to have created a substantial delay in returning to work in-person after childbirth, and allowed recently postpartum mothers to have greater control over their schedules. In one recent study, this paradigm shift was found to increase breastfeeding frequency and delay weaning for employed mothers [29].

We found no association between returning to work part-time and breastfeeding cessation by 3 months compared to nonworking women, which contrasts with research showing that women who returned to work full-time before 3 months had a higher odds of stopping breastfeeding by 3 months compared to women who did not work [19]. Our findings suggest that employment itself may not be the main barrier to improving breastfeeding outcomes among employed women, but instead, the more important factor may be having a flexible work schedule. Breastfeeding is time-intensive and both mentally and physically demanding, and a flexible work schedule can provide the time and space needed to accommodate breastfeeding [10, 19, 30]. The overwhelming departure from traditional workplace settings during the pandemic is likely to have increased schedule and time flexibility, which improved breastfeeding outcomes for employed women who would not otherwise have been able to breastfeed due to work constraints.

We found variations in sub-groups, with women who were not married, younger, less educated, and who did not receive any paid maternity leave less likely to continue breastfeeding if they took a shorter maternity leave than if they took a longer maternity leave. Given that health and economic issues related to COVID-19 were not experienced equally across the population, it is likely that stressors of the pandemic intensified existing socioeconomic and class disparities [31]. Social, health, and economic gaps widened between married and unmarried people while benefits experienced by married people accumulated between April and December 2020 [32].

Our findings suggest that being legally married resulted in economic advantages during the pandemic given the benefits of having a second income during maternity leave. Women who were not legally married may not have experienced the same financial support from a partner to whom they were not legally married during maternity leave. Other studies have found that married individuals experienced significant economic advantages during the pandemic relating to income, food sufficiency, and issues with housing or medical care compared to legally married people [32]. Being in a registered marriage during the COVID-19 pandemic reduced the adverse effects of employment disruptions [33]. Unfortunately, the PRAMS questionnaire only included questions about marriage as a binary variable with one category being legally married and the other category as all other relationship statuses, so we are not able to distinguish among multiple categories of partnerships.

Mental health is a known factor linked with breastfeeding outcomes, and maternal depression may increase the likelihood of breastfeeding cessation [34]. Between April-December 2020, both non legally-married adults and adults with lower education levels were more likely to experience higher depressive symptoms and health problems compared to legally married and more highly educated adults [32]. These findings correspond to other studies that have reported health and economic advantages for married women and highly educated women during the pandemic compared to women who were not married or less educated [32, 33, 35]. Thus, structural disadvantages experienced by women who were non-married or less educated during the COVID-19 pandemic may have accelerated breastfeeding cessation.

Our findings suggest that the ability to take paid leave may have minimized the differences in breastfeeding prevalence at 2 and 3 months between shorter and longer maternity leave lengths during the COVID-19 pandemic, possibly explained by the type of occupations held by those who qualified for any paid leave and the mechanisms by which these women were shielded from the economic strain of the pandemic [31]. Unfortunately, we do not have data on type of employment for women in our study, although future research could examine this question.

A key strength of this study is that the natural experiment of the COVID pandemic allowed us to shed light on the importance of physical proximity in the initiation and support of continued breastfeeding by using data from pre-pandemic and post-pandemic time periods. Additional strengths of this study include using a robust dataset from women with a recent live birth during the COVID-19 pandemic, the capability to adjust for multiple confounders, and the ability to replicate analyses conducted by another researcher using the same data source during a different timeframe to compare trends [16]. Our analysis relies on population-average measures, which are considered relevant for public health. PRAMS uniquely contains data from before and during the pandemic relating to breastfeeding behaviors, maternal employment, and maternity leave that we were able to incorporate into our study. Although the exact mechanisms are unclear, the conditions of the pandemic may have protected the breastfeeding relationship and minimized differences in breastfeeding initiation and duration between women who took less than 3 months of maternity leave compared to 3 months or more of maternity leave.

In spite of these strengths, our analysis has some limitations. We did not have data on the respondent’s work schedules (part-time vs. full-time) or occupations, both of which could be associated with breastfeeding outcomes. We also lacked data on the amount of pay received during maternity leave or the sources of paid leave that were utilized (vacation time, sick time, employer paid, etc.), which may have contributed to the amount of maternity leave length taken and breastfeeding outcomes. Our findings were limited to the eight PRAMS sites that included employment questions on their questionnaire and met our selection criteria, and therefore our results may not necessarily generalizable to other sites. The possibility of recall bias is a possible limitation but was also likely low given that 61% of respondents returned the PRAMS survey within 4 months of delivery, and 67% of respondents were still breastfeeding at the time they returned the survey, which suggests that the accuracy of reported breastfeeding was relatively high.

In sum, our results suggest that the conditions of the COVID-19 pandemic may have minimized differences in breastfeeding outcomes between women who took shorter maternity leaves and women who took longer maternity leaves and increased overall breastfeeding rates for employed women. Our findings suggest that the COVID-19 pandemic created an environment that protected breastfeeding for women who were older, highly educated, married and who received paid leave, and negatively affected breastfeeding outcomes for women who faced economic and social disadvantages within the United States. Social distancing and workplace flexibility may have created conditions that allowed employed mothers to be physically present with their babies longer than they would have been had they needed to return to work prior to the pandemic. Future research could explore whether post-pandemic conditions policies relating to working from home are associated with breastfeeding.

The findings from our study suggest that extended physical contact with a baby after delivery may help minimize differences in breastfeeding outcomes by maternity leave length. The results of this study point to the necessity of developing and implementing policies and programs that provide extended, paid maternity leave as well as flexible work schedules and work from home opportunities during the first year postpartum for women of all occupations. These types of reforms would help support and sustain breastfeeding by allowing mothers to maintain physical contact with their babies, and also by allowing for breastfeeding mothers to have greater financial security. Maternity leave reform and flexible work schedules for employed mothers are urgently needed, and the findings from this research could support these policy changes. Conducting a similar analysis in the post-pandemic context to evaluate the impact of workplace changes—particularly the sustained prevalence of remote work—on breastfeeding duration would be a great opportunity for future research.

## Supplementary Information

Below is the link to the electronic supplementary material.

Supplementary Material 1

## Data Availability

All analyses were conducted using PRAMS data, which are publicly available: https://www.cdc.gov/prams/php/data-research/index.html.
