# Supplementary material for: Maternity leave and breastfeeding during COVID: a cross-sectional study
Source: Int Breastfeed J. 2025 Nov 19;20:88. doi: 10.1186/s13006-025-00779-5 (PMC12628605; doi:10.1186/s13006-025-00779-5)
Supplement: Supplementary file 1 — Supplementary Material 1 [file 13006_2025_779_MOESM1_ESM.docx]

***Additional Files***

**Interaction Tables**

**Additional File 1.** Prevalence and prevalence ratio of breastfeeding initiation and breastfeeding at 1, 2, and 3 months by leave length stratified by marital status among women with a recent live birth who were employed during and after pregnancy– Pregnancy Risk Assessment Monitoring System, 8 sites – 2020-2021

|  | **Total^a^** | **Married** | | **Not Married** | | ***P* value^g^** |
| --- | --- | --- | --- | --- | --- | --- |
|  |  | **% (95% CI)^b,c^** | **APR (95% CI) ^c,d,e^** | **% (95% CI)^c, d^** | **APR (95% CI)^c,d,e^** |  |
| *Initiated breastfeeding =3,683* | | | | | | |
| *Leave length* |  |  |  |  |  | 0.265 |
| <3 months | 2,119 | 92.7 (90.7, 94.7) | 0.99 (0.96, 1.03) | 90.7 (87.8, 93.7) | 0.96 (0.92, 1.00) |  |
| ≥3 months | 1,564 | 93.3 (90.8, 95.8) | Ref (1.00) | 94.1 (91.4, 96.9) | Ref (1.00) |  |
| *Breastfeeding at 1 month=3,683* | | | | | | |
| *Leave length* |  |  |  |  |  | **0.018** |
| <3 months | 2,119 | 86.9 (84.5, 89.4) | 1.00 (0.96, 1.05) | 78.5 (74.3, 82.8) | **0.91 (0.84, 0.97)** |  |
| ≥3 months | 1,564 | 86.8 (83.6, 90.0) | Ref (1.00) | 86.5 (82.2, 90.9) | Ref (1.00) |  |
| *Breastfeeding at 2 months=3,683* | | | | | | |
| *Leave length* |  |  |  |  |  | **0.0002** |
| <3 months | 2,119 | 80.5 (77.7, 83.4) | 1.02 (0.96, 1.08) | 67.2 (62.2, 72.3) | **0.84 (0.76, 0.91)** |  |
| ≥3 months | 1,564 | 78.8 (75.2, 82.4) | Ref (1.00) | 80.4 (75.1, 85.6) | Ref (1.00) |  |
| *Breastfeeding at 3 months= 3,026 ^f^* | | | | | | |
| *Leave length* |  |  |  |  |  | 0.056 |
| <3 months | 1,704 | 77.3 (74.1, 80.6) | 1.03 (0.96, 1.10) | 63.5 (57.8, 69.4) | 0.90 (79.2, 1.02) |  |
| ≥3 months | 1,322 | 74.9 (71.0, 78.8) | Ref (1.00) | 70.3 (63.4, 77.1) | Ref (1.00) |  |

^a^ Unweighted sample size

^b^ Weighted % (95% CI)

^c^ Adjusted for type of leave, length of leave, maternal race, maternal age, maternal education, marital status, household income by federal poverty level, parity, gestational age at birth, timing of survey completion, and PRAMS site

^d^ We created separate survey-weighted multivariable logistic regression models to examine the associations with any breastfeeding at 1, 2, and 3 months by marital status between women who reported less than 3 months of maternity leave and those who reported 3 or more months of maternity leave. The reference group was women who took 3 months or more of maternity leave.

^e^ Marginal (averaged) prevalence ratios based on a logistic regression model

^f^ Sample was restricted to those women who had completed their PRAMS survey at or after 3 months after giving birth

^g^ *P* value based on the Wald test of nonlinear combinations for an interaction between leave length and marital status

**Additional File 2.** Prevalence and prevalence ratio of breastfeeding initiation and breastfeeding at 1, 2, and 3 months by leave length stratified by maternal education level among women with a recent live birth who were employed during and after pregnancy– Pregnancy Risk Assessment Monitoring System, 8 sites – 2020-2021

|  | **Total^a^** | **High school or less** | | **Some college or Associate degree** | | **Bachelor’s degree or higher** | | ***P* value*^g^*** |
| --- | --- | --- | --- | --- | --- | --- | --- | --- |
|  |  | **% (95% CI)^b,c^** | **APR**  **(95% CI) ^c,d,e^** | **% (95% CI)^b,c,^** | **APR**  **(95% CI)^c,d,e^** | **% (95% CI)^b,c^** | **APR**  **(95% CI)^c,d,e^** |  |
| *Initiated breastfeeding, n=3,683* | | | | | | | | |
| *Leave length* |  |  |  |  |  |  |  | 0.356 |
| <3 months | 2,119 | 85.4 (79.5, 91.2) | 0.95 (0.88, 1.03) | 90.4 (86.5, 94.3) | 0.97 (0.92, 1.02) | 96.9 (95.6, 98.1) | 1.00 (0.98, 1.02) |  |
| ≥3 months | 1,564 | 89.6 (83.5, 95.7) | Ref (1.00) | 93.2 (89.3, 97.1) | Ref (1.00) | 97.1 (95.5, 98.6) | Ref (1.00) |  |
| *Breastfeeding at 1 month, n=3,683* | | | | | | | | |
| *Leave length* |  |  |  |  |  |  |  | 0.184 |
| <3 months | 2,119 | 73.5 (66.3, 80.7) | 0.93 (0.81, 1.04) | 76.9 (71.4, 82.4) | 0.92 (0.83, 1.01) | 91.9 (89.8, 94.0) | 1.00 (0.96, 1.03) |  |
| ≥3 months | 1,564 | 79.1 (70.7, 87.5) | Ref (1.00) | 83.5 (77.2, 89.8) | Ref (1.00) | 92.2 (89.5, 94.9) | Ref (1.00) |  |
| *Breastfeeding at 2 months, n=3,683* | | | | | | | | |
| *Leave length* |  |  |  |  |  |  |  | **0.009** |
| <3 months | 2,119 | 58.1 (50.0, 66.3) | **0.82 (0.68, 0.96)** | 64.3 (57.9, 70.7) | 0.89 (0.77, 1.01) | 87.4 (84.8, 90.0) | 1.01 (0.97, 1.06) |  |
| ≥3 months | 1,564 | 71.2 (61.7, 80.6) | Ref (1.00) | 72.4 (64.9, 79.9) | Ref (1.00) | 86.2 (82.7, 89.6) | Ref (1.00) |  |
| *Breastfeeding at 3 months, n= 3,026 ^f^* | | | | | | | | |
| *Leave length* |  |  |  |  |  |  |  |  |
| <3 months | 1,704 | 56.8 (47.9, 65.7) | 0.95 (0.74, 1.15) | 59.8 (52.8, 66.9) | 0.92 (0.77, 1.08) | 84.3 (81.0, 87.5) | 1.02 (0.96, 1.09) | 0.404 |
| ≥3 months | 1,322 | 60.0 (49.0, 71.1) | Ref (1.00) | 64.9 (56.5, 73.4) | Ref (1.00) | 82.3 (78.3, 86.2) | Ref (1.00) |  |
|  |  |  |  |  |  |  |  |  |

^a^ Unweighted sample size

^b^ Weighted % (95% CI)

^c^ Adjusted for type of leave, length of leave, maternal race, maternal age, maternal education, marital status, household income by federal poverty level, parity, gestational age at birth, timing of survey completion, and PRAMS site

^d^ We created separate survey-weighted multivariable logistic regression models to examine the association with any breastfeeding at 2 months by maternal education level between women who reported less than 3 months of maternity leave and those who reported 3 or more months of maternity leave. The reference group was women who took 3 months or more of maternity leave.

^e^ Marginal (averaged) prevalence ratios based on a logistic regression model

*^f^* Sample was restricted to those women who had completed their PRAMS survey at or after 3 months after giving birth

^g^ *P* value based on the Wald test of nonlinear combinations for an interaction between leave length and maternal education level

**Additional File 3:** Prevalence and prevalence ratio of breastfeeding initiation and breastfeeding at 1, 2, and 3 months by leave length stratified by maternal age among women with a recent live birth who were employed during and after pregnancy– Pregnancy Risk Assessment Monitoring System, 8 sites – 2020-2021

|  | **Total^a^** | **18-24** | | **25-29** | | **30-34** | | **35+** | | ***P* value*^g^*** |
| --- | --- | --- | --- | --- | --- | --- | --- | --- | --- | --- |
|  |  | **%**  **(95% CI)^b,c^** | **APR**  **(95% CI) ^c,d,e^** | **%**  **(95% CI)^b,c^** | **APR**  **(95% CI)^c,d,e^** | **%**  **(95% CI)^b,c^** | **APR**  **(95% CI)^c,d,e^** | **%**  **(95% CI)^b,c^** | **APR**  **(95% CI)^c,d,e^** |  |
| *Initiated breastfeeding, n=3,683* | | | | | | | | | | |
| *Leave length* |  |  |  |  |  |  |  |  |  | 0.046 |
| <3 months | 2,819 | 90.0 (85.0, 94.9) | 1.00 (0.92, 1.09) | 90.8 (87.9, 93.7) | 0.93 (0.90, 0.97) | 92.9 (90.3, 95.5) | 1.01 (0.96, 1.05) | 93.4 (90.2, 96.6) | 0.98 (0.94, 1.03) |  |
| ≥3 months | 1,564 | 89.5 (83.1, 96.0) | Ref (1.00) | 97.4 (95.5, 99.3) | Ref (1.00) | 92.3 (91.6, 98.3) | Ref (1.00) | 94.9 (91.6, 98.3) | Ref (1.00) |  |
| *Breastfeeding at 1 month, n=3,683* | | | | | | | | | | |
| *Leave length* |  |  |  |  |  |  |  |  |  | 0.055 |
| <3 months | 2,119 | 81.4 (75.1, 87.6) | 0.92 (0.83, 1.01) | 82.8 (79.2, 86.5) | 91.2 (85.4, 97.1) | 84.9 (81.6, 88.3) | 1.04 (0.96, 1.11) | 86.2 (81.8, 90.6) | 0.97 (0.90, 1.03) |  |
| ≥3 months | 1,564 | 88.6 (82.2, 94.9) | Ref (1.00) | 90.8 (86.6, 95.0) | Ref (1.00) | 82.0 (77.1, 86.8) | Ref (1.00) | 89.1 (84.7, 93.3) | Ref (1.00) |  |
| *Breastfeeding at 2 months, n=3,683* | | | | | | | | | | |
| *Leave length* |  |  |  |  |  |  |  |  |  | **0.027** |
| <3 months | 2,119 | 71.1 (63.0, 79.2) | **0.85 (0.73, 0.96)** | 75.3 (71.1, 79.4) | **0.90 (0.83, 0.98)** | 77.4 (73.6, 81.2) | 1.05 (0.96, 1.14) | 79.0 (74.1, 83.9) | 0.96 (0.88, 1.05) |  |
| ≥3 months | 1,564 | 84.0 (76.5,91.4) | Ref (1.00) | 83.3 (77.8, 88.8) | Ref (1.00) | 73.9 (68.6, 79.1) | Ref (1.00) | 82.0 (77.0, 87.0) | Ref (1.00) |  |
| *Breastfeeding at 3 months, n= 3,026 ^f^* | | | | | | | | | | |
| *Leave length* |  |  |  |  |  |  |  |  |  | **0.020** |
| <3 months | 1,704 | 73.0 (64.7, 82.5) | 0.90 (0.77, 1.03) | 70.5 (65.5, 75.4) | **0.90 (0.80, 0.99)** | 73.5 (69.1,78.0) | 1.12 (1.00, 1.23) | 75.9 (70.3, 81.5) | 0.99 (0.89, 1.09) |  |
| ≥3 months | 1,322 | 81.0 (72.4, 89.6) | Ref (1.00) | 78.7 (72.5, 84.8) | Ref (1.00) | 65.9 (60.4, 71.5) | Ref (1.00) | 76.8 (71.0, 82.5) | Ref (1.00) |  |

^a^ Unweighted sample size

^b^ Weighted % (95% CI)

^c^ Adjusted for type of leave, length of leave, maternal race, maternal age, maternal education, marital status, household income by federal poverty level, parity, gestational age at birth, timing of survey completion, and PRAMS site

^d^ We created separate survey-weighted multivariable logistic regression models to examine the association with any breastfeeding at 1 month by maternal age between women who reported less than 3 months of maternity leave and those who reported 3 or more months of maternity leave. The reference group was women who took 3 months or more of maternity leave.

^e^ Marginal (averaged) prevalence ratios based on a logistic regression model

*^f^* Sample was restricted to those women who had completed their PRAMS survey at or after 3 months after giving birth

*^g^* *P* value based on the Wald test of nonlinear combinations for an interaction between leave length and maternal age

**Additional File 4: CDC PRAMS Stata Protocol**

CDC PRAMS requires the use of the following statement when using Stata to implement the PRAMS Sample Design. We followed the protocol and used this code in our Stata analysis to account for the PRAMS weighting and survey design:

svyset _*n [pweight=wtanal], strata(sud*_nest) fpc(totcnt)

Human Ethics and Consent to Participate declarations: not applicable.

No funding was received for this study.
